# Predicting impinging osteophytes based on pre-operative simulation results to guide arthroscopic débridement for elbow osteoarthritis

**DOI:** 10.1016/j.jseint.2026.101667

**Published:** 2026-02-11

**Authors:** Ko Temporin, Yusuke Yamamoto, Yuji Miyoshi, Satoshi Miyamura, Keiichiro Oura, Kozo Shimada

**Affiliations:** a Center of Hand and Trauma Surgery, Japan Community Healthcare Organization Osaka Hospital, Osaka, Japan; b Department of Orthopaedic Surgery, Japan Community Healthcare Organization Osaka Hospital, Osaka, Japan; c Department of Orthopedics and Rehabilitation Medicine, Faculty of Medical Science, University of Fukui, Fukui, Japan; d Department of Orthopaedic Surgery, Osaka University, Graduate School of Medicine, Suita, Japan; e Department of Orthopaedic Surgery, Osaka Police Hospital, Osaka, Japan

**Keywords:** Arthroscopy, Computed tomography, Elbow osteoarthritis, Osteophyte, Range of motion, Simulation

## Abstract

**Background:**

In arthroscopic surgery for elbow osteoarthritis, decisions regarding the size and location of osteophytes to be removed often depends on the surgeon's experience. The purpose of this study was to predict impinging osteophytes based on pre-operative simulation results.

**Methods:**

In total, 92 elbows with osteoarthritis were examined. Computed tomography was performed in maximum extension, 90°, and maximum flexion positions. Bone models were constructed and the rotational axes were calculated. The forearm bones were rotated around these axes to simulate the 0° and 140° positions, with the overlapping regions identified as impinging osteophytes. The sizes of the osteophytes were measured, and the relationship between osteophyte size and pre-operative range of motion was investigated.

**Results:**

For the 0° simulated position, sizes of impinging osteophytes were 6.4 ± 0.4 mm at the posteromedial humeroulnar joint, 4.2 ± 0.3 mm at the posterolateral humeroulnar joint, and 2.2 ± 0.2 mm at the posterior capitellum. Osteophyte size was negatively correlated with the extension at the posteromedial humeroulnar joint (R = −0.48, Y = −0.16X + 2.52). For the 140° simulated position, sizes of osteophytes were 6.7 ± 0.4 mm at the anterior humeroulnar joint and 2.0 ± 0.2 mm at the radial fossa. Osteophyte sizes were negatively correlated with flexion (R = −0.45, Y = −0.14X + 23.23 at the anterior humeroulnar joint; R = −0.46, Y = −0.08X + 11.20 at the radial fossa).

**Conclusion:**

Osteophytes are associated with the pre-operative range at the posteromedial humeroulnar joint, anterior humeroulnar joint, and radial fossa. These results can be used as an index to determine the osteophytes to be removed during arthroscopic surgery for elbow osteoarthritis.

Elbow osteoarthritis is characterized by impingement pain and limited range of motion, for which arthroscopic surgery is widely performed. Removal of impinging osteophytes, as well as free and loose bodies, helps improve impingement pain and range of motion.^1^^,^^3^^,^^6^^,^^9^^,^^10^

Several methods have been reported for determining which areas of osteophytes should be removed. Some authors have advocated removing the impinging lesions identified during arthroscopy.^7^^,^^8^^,^^11^ Moreover, pre-operative simulation using computed tomography (CT) data has been reported as a tool for precise surgical planning. Some studies have utilized CT data obtained in a single position to create a virtual rotational axis based on bony morphology to simulate flexion and extension motion.^14^^,^^20^ Miyake et al^13^ described a method for calculating rotational axes using CT data acquired in 3 positions, allowing more accurate motion simulation. These simulations are useful during arthroscopic surgery; however, their application is limited because specialized software, which is available only at a limited number of institutions and demands considerable skills to use, is required. If the size and location of impinging osteophytes could be predicted pre-operatively, this would be beneficial for arthroscopic procedures in facilities where such simulations are unavailable.

In this study, we reviewed our institution's simulation results to investigate the relationships between pre-operative conditions and the size and location of impinging osteophytes. We hypothesized that the characteristics of impinging osteophytes can be predicted based on the pre-operative range of motion.

## Materials and methods

### Participants

This retrospective study was approved by the institutional review board of our hospital, and written informed consent was obtained from all participants.

**Figure 1:**
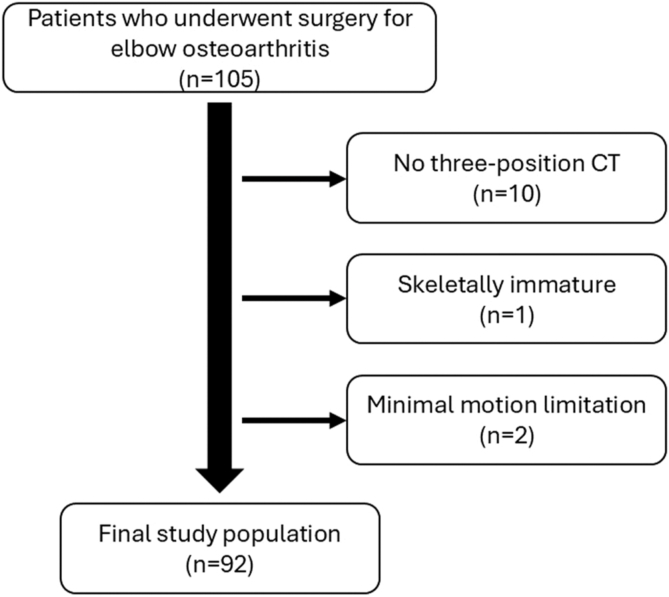
Flow diagram of patient selection. *CT*, computed tomography.

Patients who underwent arthroscopic surgery for elbow osteoarthritis between 2009 and 2024 were enrolled. The inclusion criteria were as follows: availability of pre-operative three-position CT, completion of pre-operative simulation, and performance of arthroscopic surgery for elbow osteoarthritis aimed at improving elbow motion. Patients who were skeletally immature or had minimal motion limitation—defined as flexion >140° and extension >0°—were excluded. A total of 92 elbows were included in this study (Fig. 1). There were no cases of post-traumatic arthritis involving the displacement of the articular surfaces or severe deformity.

### Pre-operative simulation

A helical CT scan (Revolution CT; GE Healthcare, WI, USA) was performed in 3 positions: maximum extension, 90°, and maximum flexion. CT was acquired at low radiation doses to minimize exposure.^15^ The Digital Imaging and Communications in Medicine data were imported into commercially available software (BoneViewer; Orthree, Osaka, Japan). Three-dimensional bone models of the humerus, ulna, and radius were reconstructed for each of the 3 positions. Any loose body attached to the bone and moving synchronously with it was modeled as part of the bone.

**Figure 2:**
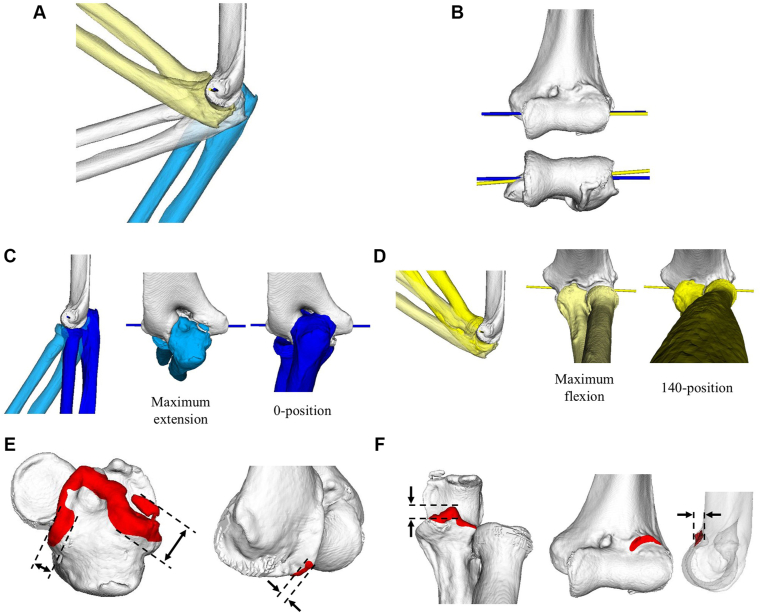
Pre-operative simulation. (**A**) Bone models in 3 positions. Light blue: maximum extension; white: 90°; *light yellow*: maximum flexion. (**B**) Rotational axes in extension (*yellow*) and flexion (*blue*) ranges. (**C**) Simulation of the 0° position. The forearm bones were rotated around the extension axis from maximum extension (*light blue*) to the 0° position (*blue*). (**D**) Simulation of the 140° position. The forearm bones were rotated around the flexion axis from maximum flexion (*light yellow*) to the 140° position (*yellow*). (**E** and **F**) Simulation of impinging osteophytes in the 0° (**E**) and 140° (**F**) positions. The *red* regions represent osteophytes requiring removal. Arrows indicate the size.

The humeral models from the 3 positions were superimposed (Fig. 2*A*). The movement of the ulna and radius between positions was calculated. Using these data, the extension axis (between maximum extension and 90°) and the flexion axis (between maximum flexion and 90°) were calculated (Fig. 2*B*).

A simulated 0° position was generated by moving the ulna and radius from the maximum extension position toward further extension around the extension axis, representing full extension (Fig. 2*C*). Similarly, a 140° position was generated by moving the ulna and radius from the maximum flexion position toward further flexion around the flexion axis, representing full flexion (Fig. 2*D*). Overlapping regions between the humerus and the ulna/radius in the simulated 0° and 140° positions were defined as impinging osteophytes that should be removed to restore normal elbow motion. The maximum width of impinging osteophytes was measured in specific anatomical regions: the medial olecranon for the posteromedial humeroulnar joint, lateral olecranon for the posterolateral humeroulnar joint, posterior capitellum in the 0° position, coronoid process for the anterior humeroulnar joint, and anterior radial fossa in the 140° position (Fig. 2 *E*, *F*). When the osteophytes at the medial and lateral sides of the olecranon were continuous, they were measured at the medial and lateral regions of the olecranon tip, respectively.

To evaluate the movement of the rotational axes, changes in the flexion axis relative to the extension axis were analyzed. Angular differences were measured in the varus—valgus direction in the coronal plane and in the internal—external rotation direction in the axial plane.

### Surgery

Surgery was performed under general anesthesia with the patient in the prone position. Initially, a 4.0-mm 45° arthroscope was inserted into the anterior joint space via the anteromedial and anterolateral portals. After synovectomy, osteophytes and loose bodies in the coronoid process and coronoid fossa were removed. The size and location of resection were determined according to the pre-operative simulation. The size of the osteophyte was measured arthroscopically in comparison to the size of surgical instruments, such as a 4-mm abrader burr and bone chisel. The total amount of bone resection corresponded to the simulated plan. For example, osteophytes of the anterior humeroulnar joint identified in the simulation could be resected from the coronoid process, the coronoid fossa, or both, as appropriate. At the anterior humeroradial joint, osteophytes of the radial fossa were removed in the same manner.

Next, the posterior and posterolateral portals were used to access the posterior humeroulnar joint, where osteophytes in the olecranon and olecranon fossa were resected. For patients with posterior humeroradial impingement, 2 parallel direct lateral portals were established to resect osteophytes from the posterior capitellum. In patients with severe flexion limitation, a small incision was made at the posteromedial site to release the ulnar nerve and dissect the posterior oblique ligament.^19^ In cases complicated by cubital tunnel syndrome, anterior transposition of the ulnar nerve was additionally performed.

A suction drain was placed to remove intra-articular hematoma, and after it was removed on post-operative day 1, gentle range of motion exercises were initiated under the supervision of an occupational therapist.

### Post-operative evaluation

In 13 cases in which CT evaluation was performed within 6 months post-operatively, post-operative bone models were created and compared with the pre-operative simulations. The post-operative bone models were superimposed onto the pre-operative bone models to evaluate the presence and size of residual osteophytes.

To assess the clinical utility of the simulation, surgical outcomes were evaluated. In patients with a minimum follow-up of 1 year, flexion and extension angles were reviewed at the pre-operative stage, at 1 year post-operatively, and at the final follow-up. Flexion and extension angles were measured using a goniometer in the neutral position of pronation and supination. The Mayo Elbow Performance Score was assessed pre-operatively and at the last follow-up.

### Statistical analysis

For the size measurements, inter-rater and intrarater reliability was assessed in a randomly selected sample of 40 cases. Inter-rater reliability was evaluated using the intraclass correlation coefficient (ICC) based on a two-way random-effects model with absolute agreement for single measurements (ICC(2,1)). All 40 cases were independently assessed by the same 2 raters, who were considered representative of a larger population of raters. Intrarater reliability was assessed using a 1-way random-effects model for single measurements (ICC(1,1)). One rater repeated the assessments of the same 40 cases after a 2-week interval.

Differences in the size of the osteophytes between the regions were investigated using the Mann–Whitney U or Kruskal–Wallis tests. Correlation analysis was performed to determine the relationship between the range of motion and size of the osteophytes. Regression equations and 95% prediction intervals were calculated. Changes in the range of motion were estimated by Friedman test.

An a priori power analysis was conducted using G∗Power (version 3.1.9.7; Heinrich Heine University Düsseldorf, Düsseldorf, Germany). For the Mann–Whitney U test, assuming an effect size of d = 1.56, a significance level of α = 0.05, and a statistical power of 0.80, the minimum required sample size was estimated to be 8 participants per region. For the Kruskal–Wallis test, because G∗Power does not provide a direct option for the test, a conservative approximation of the required sample size was estimated based on a 1-way analysis of variance. Assuming an effect size of f = 0.25, a significance level of α = 0.05, and a power of 0.80, the minimum required total sample size was estimated to be 159 (53 per region). The final analysis included a total of 92 elbows, which exceeded the required sample size.

## Results

### Study patients

Ninety-two elbows from 90 patients (82 men and 8 women) were included in this study. The mean age at surgery was 50 ± 15 (range, 14–78) years. Pre-operative range of motion averaged 119 ± 12° (range, 75–145°) in flexion, −24 ± 10° (range, −45° to −5°) in extension, 75 ± 11° (range, 50–90°) in pronation, and 82 ± 11° (range, 50–90°) in supination. The demographic data of the 92 elbows are summarized in Table I. Causes of postarthritic osteoarthritis were arthritis during childhood and pseudogout arthritis.

**Table I:** Patient demographics.

Variable	Total (N = 90 patients, 92 elbows)
Age, yr	50 ± 15
Sex	
Male	82
Female	8
Affected side	
Right	78
Left	14
Cause of osteoarthritis	
Idiopathic	10
Sport	55
Heavy labor	16
Traumatic	9
Postarthritis	2

### Change of rotational axis

**Figure 3:**
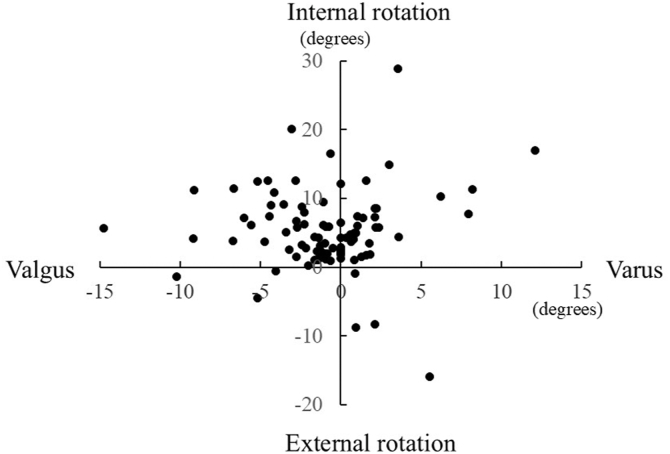
Change in rotational axes.

In the coronal plane, the flexion axis shifted an average of 0.9 ± 0.4° toward the valgus direction (range, 12.0° varus to 14.8° valgus) relative to the extension axis. Overall, 51 elbows showed a valgus shift, 10 showed no change, and 31 showed a varus shift. In the axial plane, the flexion axis shifted an average of 5.2 ± 0.6° toward internal rotation (range, 28.8° internal to 15.9° external rotation). A total of 7 elbows showed a shift toward external rotation, while 85 showed a shift toward internal rotation (Fig. 3).

### Osteophytes in simulation

**Figure 4:**
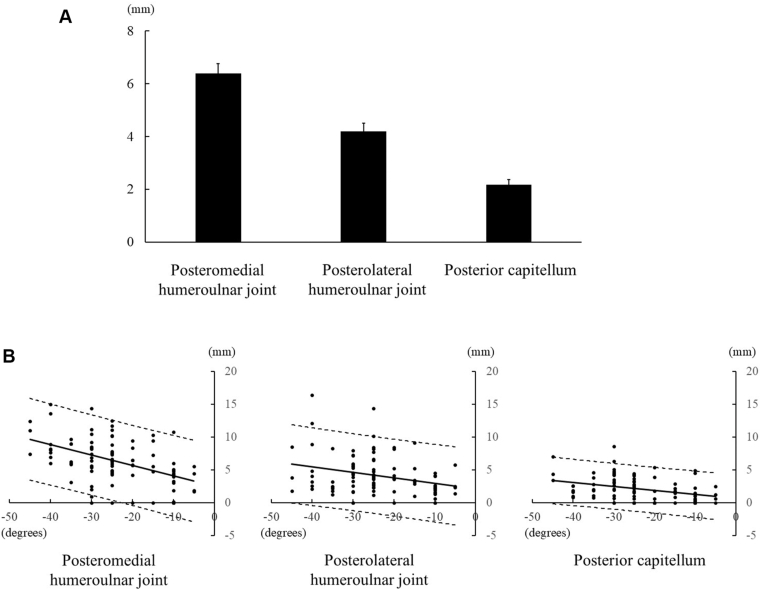
Osteophytes in the 0° position simulation. (**A**) Size of the osteophytes. (**B**) Relationship between pre-operative extension and the size of osteophytes. *Solid line*: regression equation; *dotted lines*: Upper and Lower limits of the 95% prediction interval.

In the extension simulation at the 0° position, impinging osteophytes measured 6.4 ± 0.4 mm at the posteromedial humeroulnar joint, 4.2 ± 0.3 mm at the posterolateral humeroulnar joint, and 2.2 ± 0.2 mm at the posterior capitellum. The osteophyte sizes differed significantly between regions (*P* < .01 for all comparisons: posteromedial vs. posterolateral humeroulnar joint, posterior capitellum vs. posterolateral humeroulnar joint, and posterior capitellum vs. posteromedial humeroulnar joint) (Fig. 4*A*). At the posteromedial humeroulnar joint, osteophyte size was negatively correlated with extension (R = −0.48, *P* < .001, Y = −0.16X + 2.52) (Fig. 4*B*, Table II). The upper and lower limits of the 95% prediction interval for each region are shown in Fig. 4*B*.

**Table II:** Results of correlation and regression analysis.

Region	R	*P* value	Regression line	
			Slope	Intercept
Posteromedial humeroulnar joint	−0.48	<.001	−0.16	2.52
Posterolateral humeroulnar joint	−0.29	.006	−0.08	2.16
Posterior capitellum	−0.34	.001	−0.06	0.72
Anterior humeroulnar joint	−0.45	<.001	−0.14	23.23
Radial fossa	−0.46	<.001	−0.08	11.20

**Figure 5:**
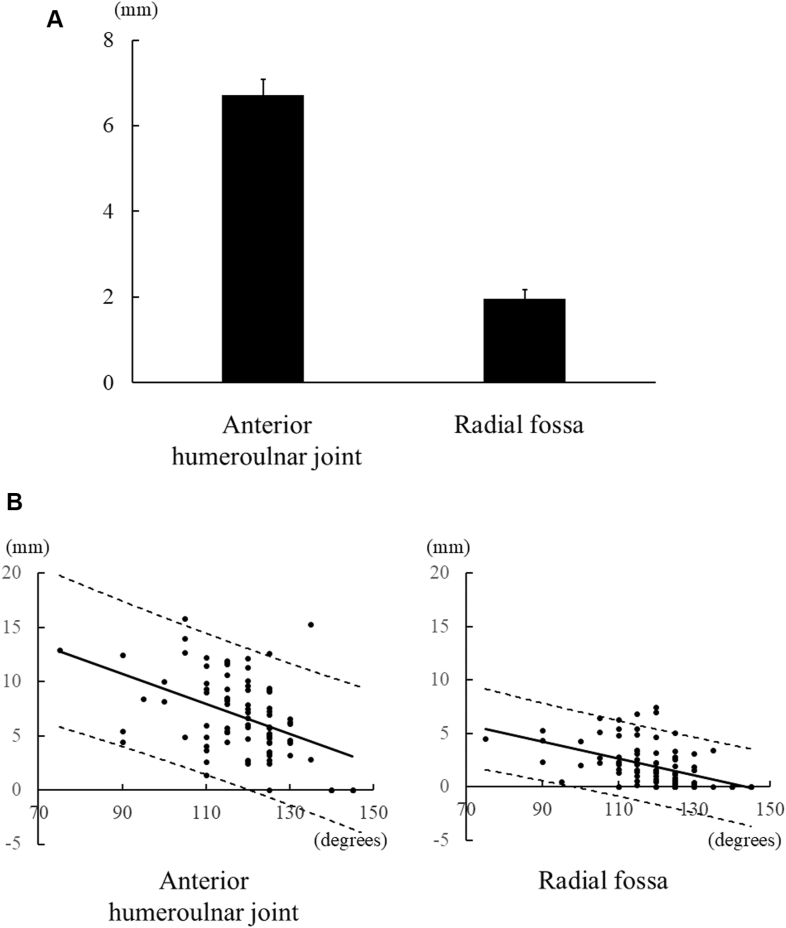
Osteophytes in the 140° position simulation. (**A**) Size of the osteophytes. (**B**) Relationship between pre-operative flexion and the size of osteophytes. *Solid line*: regression equation; *dotted lines*: Upper and Lower limits of the 95% prediction interval.

In the flexion simulation at the 140° position, impinging osteophytes measured 6.7 ± 0.4 mm at the anterior humeroulnar joint and 2.0 ± 0.2 mm at the radial fossa, with a significant difference between regions (*P* < .01) (Fig. 5*A*). Osteophyte size correlated with flexion at both sites (R = −0.45, *P* < .001, Y = −0.14X + 23.23 at the anterior humeroulnar joint; R = −0.46, *P* < .001, Y = −0.08X + 11.20 at the radial fossa) (Fig. 5*B*, Table II). The upper and lower limits of the 95% prediction interval for each region are shown in Fig. 5*B*.

### Inter-rater and intrarater reliability

The inter-rater reliability was excellent, with an ICC(2,1) of 0.99 (95% confidence interval [CI]: 0.992–0.998) at the posteromedial humeroulnar joint, 0.96 (95% CI: 0.958–0.988) at the posterolateral humeroulnar joint, 0.99 (95% CI: 0.988–0.997) at the posterior capitellum, 0.99 (95% CI: 0.992–0.998) at the anterior humeroulnar joint, and 0.99 (95% CI: 0.988–0.997) at the radial fossa. The intrarater reliability was also excellent, with an ICC(1,1) of 0.99 (95% CI: 0.988–0.996) at the posteromedial humeroulnar joint, 0.98 (95% CI: 0.965–0.990) at the posterolateral humeroulnar joint, 0.99 (95% CI: 0.975–0.993) at the posterior capitellum, 0.99 (95% CI: 0.986–0.996) at the anterior humeroulnar joint, and 0.98 (95% CI: 0.969–0.991) at the radial fossa.

### Post-operative evaluation

According to the post-operative bone models, the residual osteophytes at all sites were less than 1 mm in size in all cases. The residual osteophytes measured 0.4 ± 0.1 mm at the posteromedial humeroulnar joint, 0.4 ± 0.1 mm at the posterolateral humeroulnar joint, 0.2 ± 0.1 mm at the posterior capitellum, 0.3 ± 0.1 mm at the anterior humeroulnar joint, and 0.3 ± 0.1 mm at the radial fossa.

A total of 58 elbows were followed for a minimum of 1 year (mean, 26 ± 21 months; range, 12–97 months). Flexion significantly improved from 118 ± 13° pre-operatively to 128 ± 13° at 1 year (*P* < .01) and was maintained at 127 ± 14° at the final follow-up (*P* < .01 compared with pre-operative). Extension improved from −25 ± 11° pre-operatively to −16 ± 9° at 1 year (*P* < .01) and −17 ± 12° at the final follow-up (*P* < .01 compared with pre-operative).

Mayo Elbow Performance Score improved from 74 ± 8 points pre-operatively (4 elbows poor, 19 fair, 35 good) to 89 ± 11 points at the final follow-up (1 poor, 5 fair, 22 good, 30 excellent) (*P* < .01). In 1 poor case, the range of motion initially improved at 1 year but subsequently declined due to recurrent osteophyte formation, necessitating revision surgery.

## Discussion

In this study, the sizes of impinging osteophytes increased as the limitation of range of motion became more severe. The size exhibited a linear relationship with the range of motion at the posteromedial humeroulnar joint, anterior humeroulnar joint, and radial fossa, and the regression equation and 95% prediction interval could be calculated. On referencing the 95% prediction interval based on the pre-operative range of motion, the size of the osteophyte to be removed could be predicted in most patients. These results can be used as an index for determining how much of an osteophyte should be removed during arthroscopic surgery for elbow osteoarthritis.

Osteophyte resection for elbow osteoarthritis is commonly performed according to the surgeons' clinical recognition.^16^ Pre-operative evaluation using CT and arthroscopic observation are utilized to identify impinging osteophytes,^9^ and impinging osteophytes should be removed until no impingement remains.^7^^,^^8^^,^^11^ However, the procedure often depends on the surgeon's experience, making it difficult to standardize surgical techniques. Arthroscopy provides a limited field of view, preventing comprehensive visualization. Thus, there is a possibility that osteophytes may be overlooked. To resolve this problem, pre-operative simulation has been proposed.^13^, ^14^, ^15^^,^^20^ Such simulation can assist surgeons by indicating the size and location of osteophytes that need to be removed.

Pre-operative simulations for arthroscopic surgery of elbow osteoarthritis have been reported previously. Nishiwaki et al used CT data to simulate elbow motion by modeling the capitellum and trochlear groove as a sphere and a circle, respectively, and creating a virtual rotational axis by connecting their centers. The ulna and radius were rotated around this axis, and the overlapping region was identified as the impinging osteophyte.^14^ Yamamoto et al^20^ simulated elbow motion using a virtual axis formed by connecting the centers of 2 circles within the capitellum and trochlea. Both studies were based on static CT data in a single position and did not accurately reproduce elbow flexion and extension movement. Miyake et al used CT data from 3 positions: maximum extension, 90°, and maximum flexion. Rotational axes were calculated based on the movement of the ulna and radius.^13^ Although CT in 3 positions increased radiological exposure, more precise simulation could be achieved. Shigi et al^16^ advanced this method and constructed a three-dimensionally printed model that showed impinging regions. Even in healthy elbows, the rotational axis was reported to move during flexion and extension.^2^^,^^5^ In elbow osteoarthritis, deformation at the articular surface and its edge occurs,^17^ and the axis can shift more significantly. Indeed, substantial axis changes were observed in some patients in this study. These findings support the value of three-position simulation for more precise pre-operative planning.

Undoubtedly, precise simulations are the best tool for pre-operative planning. However, simulation requires certain technical skills, such as creating bone models, setting rotational axes, and calculating impinging osteophytes. Specific devices or software are also necessary. Simulation requires time to be performed prior to surgery. Therefore, not all institutions can perform the simulation. According to the results of this study, the size of osteophytes was related to the pre-operative range of motion in the posteromedial humeroulnar joint, anterior humeroulnar joint, and radial fossa. The size and location can be predicted based on the pre-operative range of motion, and our results provide a good index for arthroscopic surgery in institutions where simulation is not available, particularly for less experienced surgeons. However, in the posterolateral humeroulnar joint and posterior capitellum, a weaker relationship was shown, and careful arthroscopic observation is needed to avoid missing impingement. Of course, individualized assessment remains crucial in each case.

The main impinging region of the posterior part was the olecranon. The size of the osteophytes tended to be larger on the medial side. Osteophytes were reported to form at the medial part of the olecranon in the early stage of osteoarthritis,^12^ and impingement was likely to occur in this region.^9^^,^^18^ The elbow has physiological valgus, and the medial side may be prone to impingement during extension. The posterior capitellum had the smallest osteophytes, but this region was reported to be often overlooked.^14^ The impinging size was greater at the humeroulnar joint than at the humeroradial joint, in consistency with a previous report.^14^

Several considerations should be noted regarding simulation. First, osteophyte resection should be judged according to the patient's background. For example, in elbow osteoarthritis associated with baseball, osteophyte formation can serve as a supportive adaptation for an unstable elbow. Medial laxity induces valgus instability, and osteophytes in the posteromedial humeroulnar joint may help support the instability. In this condition, excessive resection of osteophytes in the olecranon fossa may exacerbate valgus instability, causing medial pain.^4^ Second, simulation has potential sources of error. In patients with massive deformity of the articular surface, such as post-traumatic osteoarthritis, the rotational axis may change substantially during flexion and extension motions. In severely stiff elbows, the rotational axis can be calculated only from a very limited range of motion, creating potential error. Consequently, discrepancies may exist between the simulated axis and the actual axis. Simulation with three-position CT is a method to minimize this gap. During surgery, the simulation should be used with these points in mind, and final confirmation under arthroscopy is necessary to ensure that all impingement has been removed, especially in the posterolateral humeroulnar joint and posterior capitellum. In elbow osteoarthritis surgery, factors affecting range of motion include not only bony impingement but also soft tissue tightness. Resection of osteophytes alone does not achieve ideal improvement in range of motion.

This study had some limitations. First, various etiologies of osteoarthritis were included in this study. Although simulation results might vary depending on the etiology, the results were considered to reflect osteoarthritis as a whole. Another limitation was the small proportion of women (8 of 90 cases) included in this study. Moreover, the follow-up period to investigate the outcome was relatively short, with a minimum of 1 year. However, the outcome was examined for the assessment of the simulation, and 1 year was sufficient to investigate its usefulness. The correlations were moderately strong or weak. This is thought to be caused by significant individual variation in osteophyte formation, resulting in considerable variability. Further investigation is needed regarding the sites where osteophytes form in individual cases.

## Conclusion

In elbow osteoarthritis, the size and location of osteophytes are related to the pre-operative range of motion in the posteromedial humeroulnar joint, anterior humeroulnar joint, and radial fossa. These results can be used as an index to determine the extent to which osteophytes should be removed during arthroscopic surgery for elbow osteoarthritis. Based on these results, arthroscopic surgery can be performed confidently.

## Disclaimers:

Funding: No funding was disclosed by the authors.

Conflicts of interest: The authors, their immediate family, and any research foundation with which they are affiliated did not receive any financial payments or other benefits from any commercial entity related to the subject of this article.

## References

[1] Adams J.E., Wolff L.H., Merten S.M., Steinmann S.P. (2008). Osteoarthritis of the elbow: results of arthroscopic osteophyte resection and capsulectomy. J Shoulder Elbow Surg.

[2] Bottlang M., Madey S.M., Steyers C.M., Marsh J.L., Brown T.D. (2000). Assessment of elbow joint kinematics in passive motion by electromagnetic motion tracking. J Orthop Res.

[3] Chow H.Y., Eygendaal D., The B. (2021). Elbow arthroscopy - indications and technique. J Clin Orthop Trauma.

[4] Desmoineaux P., Carlier Y., Mansat P., Bleton R., Rouleau D.M., Duparc F. (2019). Arthroscopic treatment of elbow osteoarthritis. Orthop Traumatol Surg Res.

[5] Goto A., Moritomo H., Murase T., Oka K., Sugamoto K., Arimura T. (2004). In vivo elbow biomechanical analysis during flexion: three-dimensional motion analysis using magnetic resonance imaging. J Shoulder Elbow Surg.

[6] Guerrero E.M., Bullock G.S., Helmkamp J.K., Madrid A., Ledbetter L., Richard M.J. (2020). The clinical impact of arthroscopic vs. open osteocapsular débridement for primary osteoarthritis of the elbow: a systematic review. J Shoulder Elbow Surg.

[7] Kelly E.W., Bryce R., Coghlan J., Bell S. (2007). Arthroscopic debridement without radial head excision of the osteoarthritic elbow. Arthroscopy.

[8] Kokkalis Z.T., Schmidt C.C., Sotereanos D.G. (2009). Elbow arthritis: current concepts. J Hand Surg Am.

[9] Kroonen L.T., Piper S.L., Ghatan A.C. (2017). Arthroscopic management of elbow osteoarthritis. J Hand Surg Am.

[10] Kwak J.M., Kholinne E., Sun Y., Lim S., Koh K.H., Jeon I.H. (2019). Clinical outcome of osteocapsular arthroplasty for primary osteoarthritis of the elbow: comparison of arthroscopic and open procedure. Arthroscopy.

[11] Lim T.K., Koh K.H., Lee H.I., Shim J.W., Park M.J. (2014). Arthroscopic débridement for primary osteoarthritis of the elbow: analysis of pre-operative factors affecting outcome. J Shoulder Elbow Surg.

[12] Miyake J., Shimada K., Moritomo H., Kataoka T., Murase T., Sugamoto K. (2013). Kinematic changes in elbow osteoarthritis: in vivo and 3-dimensional analysis using computed tomographic data. J Hand Surg Am.

[13] Miyake J., Shimada K., Oka K., Tanaka H., Sugamoto K., Yoshikawa H. (2014). Arthroscopic debridement in the treatment of patients with osteoarthritis of the elbow, based on computer simulation. Bone Joint J.

[14] Nishiwaki M., Willing R., Johnson J.A., King G.J., Athwal G.S. (2013). Identifying the location and volume of bony impingement in elbow osteoarthritis by 3-dimensional computational modeling. J Hand Surg Am.

[15] Oka K., Murase T., Moritomo H., Goto A., Sugamoto K., Yoshikawa H. (2009). Accuracy analysis of three-dimensional bone surface models of the forearm constructed from multidetector computed tomography data. Int J Med Robot.

[16] Shigi A., Oka K., Tanaka H., Shiode R., Murase T. (2021). Utility of a 3-dimensionally printed color-coded bone model to visualize impinging osteophytes for arthroscopic débridement arthroplasty in elbow osteoarthritis. J Shoulder Elbow Surg.

[17] Temporin K., Miyoshi Y., Miyamura S., Shimada K. (2024). Bone deformity in sports-related elbow osteoarthritis: influence of osteochondritis dissecans of the capitellum-a cross-sectional study. Arch Orthop Trauma Surg.

[18] Wada T., Isogai S., Ishii S., Yamashita T. (2004). Débridement arthroplasty for primary osteoarthritis of the elbow. J Bone Joint Surg Am.

[19] Williams B.G., Sotereanos D.G., Baratz M.E., Jarrett C.D., Venouziou A.I., Miller M.C. (2012). The contracted elbow: is ulnar nerve release necessary?. J Shoulder Elbow Surg.

[20] Yamamoto M., Murakami Y., Iwatsuki K., Kurimoto S., Hirata H. (2016). Feasibility of four-dimensional pre-operative simulation for elbow debridement arthroplasty. BMC Musculoskelet Disord.

